# Supplementary material for: Vascular-derived SPARC and SerpinE1 regulate interneuron tangential migration and accelerate functional maturation of human stem cell-derived interneurons
Source: eLife. 2021 Apr 27;10:e56063. doi: 10.7554/eLife.56063 (PMC8099424; doi:10.7554/eLife.56063)
Supplement: Supplementary file 1. — HEK 293 and HBEC-5i cell lines were pre-treated in serum-free medium as if they were being used to collect conditioned medium. Cells were lysed and RNA was collected for bulk RNA-sequencing (RNA-seq) analysis. Top differentially expressed genes were screened by protein size and using the Gene Ontology (GO) term extracellular space in order to identify candidates listed in this table. [file elife-56063-supp1.docx]

**Table 1. Short List of Candidate Factors**

| **Gene Name** | **logFC** | **logCPM** | **p Value** | **p. adj.** | **RPKM (HEK 293)** | **RPKM (HBEC5i)** | **MWt (kDa)** | **Ensembl Gene ID** |
| --- | --- | --- | --- | --- | --- | --- | --- | --- |
| FSTL1 | 3.348778495 | 9.378637525 | 2.28E-159 | 1.04E-157 | 24.35624543 | 248.0976828 | 35 | ENSG00000163430 |
| LGALS3BP | 5.911208677 | 8.845241619 | 0 | 0 | 6.512443202 | 392.3319491 | 65.3 | ENSG00000108679 |
| SPARC | 3.256424679 | 8.781507534 | 3.04E-190 | 1.78E-188 | 23.19716423 | 221.6506905 | 34.6 | ENSG00000113140 |
| CFB | 11.40056669 | 8.663965342 | 0 | 0 | 0.014538627 | 39.76957639 | 85.5 | ENSG00000239754 |
| CTSB | 3.119837842 | 8.258669772 | 2.89E-189 | 1.69E-187 | 13.35919919 | 116.1479495 | 37.8 | ENSG00000164733 |
| QSOX1 | 3.995771978 | 8.202982311 | 4.20E-280 | 4.06E-278 | 24.87118604 | 396.9219637 | 82.6 | ENSG00000116260 |
| TFPI | 6.024901911 | 7.08173077 | 0 | 0 | 0.99054918 | 64.40604704 | 35 | ENSG00000003436 |
| SERPINE1 | 7.619715704 | 6.831020305 | 0 | 0 | 0.201111926 | 39.23604811 | 45.1 | ENSG00000106366 |
| DKK3 | 6.260818669 | 6.750817726 | 0 | 0 | 0.888760193 | 68.06841108 | 38.4 | ENSG00000050165 |
| TGFBI | 8.941228575 | 6.495778351 | 0 | 0 | 0.113467844 | 55.88575117 | 74.7 | ENSG00000120708 |
| APOL1 | 11.8270619 | 6.408852943 | 0 | 0 | 0.015709026 | 58.50588335 | 44 | ENSG00000100342 |
| CTGF | 6.195254089 | 6.343138734 | 0 | 0 | 0.932419705 | 68.32282348 | 38.1 | ENSG00000118523 |
| APOE | 4.492448201 | 6.332837218 | 9.97E-263 | 9.08E-261 | 5.723036671 | 129.094163 | 36.2 | ENSG00000130203 |
| LGALS9 | 8.49967528 | 6.294467191 | 0 | 0 | 0.258055301 | 93.79219528 | 39.5 | ENSG00000168961 |
| PTX3 | 7.311277226 | 6.241946337 | 0 | 0 | 0.480648722 | 77.56904198 | 42 | ENSG00000163661 |
| GDF15 | 8.150681704 | 6.117435331 | 0 | 0 | 0.398477527 | 115.2245066 | 34.1 | ENSG00000130513 |
| ECM1 | 8.374895102 | 5.970379436 | 0 | 0 | 0.323836508 | 110.4520464 | 60.7 | ENSG00000143369 |
| CLEC11A | 4.675928196 | 5.904033185 | 1.66E-141 | 6.46E-140 | 3.185910981 | 81.8615547 | 35.7 | ENSG00000105472 |
| METRNL | 3.148505934 | 5.836447482 | 2.76E-112 | 7.88E-111 | 5.274445292 | 46.69553244 | 34.4 | ENSG00000275031 |
| CTSZ | 4.023191837 | 5.822370192 | 1.31E-209 | 8.86E-208 | 4.671782343 | 76.03549352 | 33.9 | ENSG00000101160 |
| SERPING1 | 5.524428493 | 5.520462919 | 5.39E-278 | 5.18E-276 | 1.019382593 | 47.03615859 | 55.2 | ENSG00000149131 |
| TCN2 | 4.908840649 | 5.258207438 | 7.98E-228 | 6.04E-226 | 1.183167514 | 35.70635444 | 47.5 | ENSG00000185339 |
| PCOLCE | 3.86640965 | 5.132034764 | 1.77E-114 | 5.19E-113 | 2.27828812 | 33.37582118 | 48 | ENSG00000106333 |
